# Social and Poor Law Problems

**Published:** 1907-08-24

**Authors:** 


### SOCIAL AND POOR LAW PROBLEMS.

### PROMPT REGISTRATION AND INFANT LIFE.

Towards the end of last summer the St. Pancras Borough Council adopted tentatively a scheme for inducing parents to register the births of their children as speedily as possible by promising the father, or failing him a person attending the mother, a shilling if the birth was notified within forty-eight hours of its occurrence. The reason for this honorarium was that early notification of a birth facilitates the operation of many of the efforts now being made to secure the life and health of the babies of the poor. Attentions which later would be regarded as impertinences are accepted as suitable compliments to a "new baby" and its mother. The health visitor may call, and if she is a tactful person may give good advice and even leave a card of instructions as to the bringing up of infants, without being regarded at that interesting time as an intruder. In cases of real poverty the health visitor can provide food which may save the life of both mother and child, and in those more numerous cases where the fault lies with improper rather than insufficient feeding, sensible facts about food, baths, clothing, and the like—especially if told as personal reminiscences rather than as didactic statements—may have a useful effect. The scheme was introduced for a period of three months, and it seems that the St. Pancras parents approve of it, for the number of births notified within the time-limit was 299, and the amount paid out in fees nearly £15. Dr. Sykes, the medical officer of health for the borough, is of opinion that the plan has fulfilled the purpose for which it was intended, and the results of this early notification are so satisfactory that he considers it desirable that a law should be passed to make the registration of births within five days of their occurrence compulsory, as is the case with deaths. This would prevent the delay in registration which at present in many cases reacts so unfavourably upon the child.

### WORKMEN'S CLOTHES.

A curious case recently tried at Bristol raises a new point in the rules of public courtesy. Two colliers were summoned for entering a tramcar, and remaining there in defiance of the conductor, while in their working clothes, "they being persons whose clothing, in the opinion of the conductor, might soil or injure the cushions or the clothing of other passengers." A miners' agent was also summoned for abetting them. The men had come straight from the pit; they forced their way past the conductor, and it was on the advice of the agent that they refused to leave the car when asked to do so. The defence was that the tramway company had no right to differentiate between different classes of working men. Their by-laws permitted them to do so, but it was contended that the by-law was unfair. The Bench, however, declined to take this point of view, and each of the men was fined 25s. It seems to us a step in the right direction when the fact that a person's clothes are offensive is made a reason for excluding him from public conveyances. In such a case the worse has always the power to exclude the better, and clean people would be obliged to do without a convenience established for the benefit of all because the dirty intruded their offensive presence. It is difficult to see why English workmen of nearly all kinds invariably go about in garments which make their neighbours draw aside from them. There are many trades in which a man cannot help getting his clothes soiled, but an overall of some kind, like the blouse of the French working man, which could be removed when he left his work, would prevent him being a source of offence to others. In America the self-respect of the worker prevents him going about in dirty, soil-stained attire, and many employers provide toilet-rooms, where the men can wash and change their outer garments before going into the street. With regard to miners, it should not be impossible to provide baths at the pithead, where they could wash when they came up from their work and put on clean clothes. The practice would tend towards their own comfort and also that of their wives, whose attempts at keeping a clean home are handicapped by the arrival of the bread-winner in a state of grime that will undo the best efforts of a good wife.

### THE IRRESPONSIBLE FATHER.

Mrs. Mackirdy, better known as Miss Olive Christian Malvery, has at last said the sane true word that has long been wanting on the subject of sweating. Most writers are content to blame the employer, the capitalist, and the whole social system for the evils of underpaid work; and there is nothing so safe or so futile as such generalities. The employer, whose work is given out to be done in private homes, cannot know the conditions under which it is performed, still less can the capitalist who lends his money to a business, and the social system—is everybody and nobody! But Mrs. Mackirdy, in her book on "Baby Toilers," blames the father—the lazy, irresponsible father who thinks he has fulfilled the whole duty of man when he has earned enough for beer-money, and leaves to his wife the work of keeping up the house and feeding and clothing, as best she can, herself and the children. To do so, she takes work—any work—at any wage she can obtain, and when her own unaided exertions cannot provide money enough for rent and such food and fire as the human frame cannot exist without, the children help in the work. This is the condition of things wherever what are called "home industries" are pursued. The evils of factory work for married women are many, but it is doubtful if they are as great as those that spring from wage-earning in the home. In the factory the woman herself and her unborn child suffer; in the home the children who have come to conscious life are martyrs as great. School hours give a certain respite—for teachers, as a rule, do not expect too much from the weary little creatures who have been working before they came to school and will have to work again when they go home—but, as is natural, under such circumstances they can provide little of the teaching that may enable the children to rise above their present miserable conditions. The result is an enormous death-rate among children, and such a poor physique among the survivors that as a national asset they are practically worthless. Complaints, however, are of little use without some proposal for cure. As a first improvement we would suggest the registering as a workshop of every place where work is done for pay. The inspectors of these "workshops" would require to understand that their visits must as often as not be paid outside ordinary working hours. Secondly, much harsher treatment should be dealt out to the able-bodied "out-of-work." The little girl in the Rev. Richard Free's parish who said that her father had been out of work "all his life" probably said a truer thing than she knew. Thirdly, a great reduction of the sentimental charity which, in the desire to make things easier for the children, enables the parents to shirk their natural duties. If, finally, young women of the lower classes could be persuaded that it is better to remain unmarried than to tie oneself for life to a man who cannot or will not maintain his family, a great deal of misery might be avoided, and we should not have so many children brought into the world to struggle, half-starved, through a few suffering years, and then to die.

